# Ultrashort-range, high-frequency communication by female mice shapes social interactions

**DOI:** 10.1038/s41598-020-59418-0

**Published:** 2020-02-14

**Authors:** M. R. Warren, R. S. Clein, M. S. Spurrier, E. D. Roth, J. P. Neunuebel

**Affiliations:** Department of Psychological and Brain Sciences, University of Delaware, Newark, Delaware USA

**Keywords:** Animal behaviour, Social behaviour

## Abstract

Animals engage in complex social encounters that influence social groups and resource allocation. During these encounters, acoustic signals, used at both short and long ranges, play pivotal roles in regulating the behavior of conspecifics. Mice, for instance, emit ultrasonic vocalizations, signals above the range of human hearing, during close-range social interactions. How these signals shape behavior, however, is unknown due to the difficulty in discerning which mouse in a group is vocalizing. To overcome this impediment, we used an eight-channel microphone array system to determine which mouse emitted individual vocal signals during 30 minutes of unrestrained social interaction between a female and a single male or female conspecific. Females modulated both the timing and context of vocal emission based upon their social partner. Compared to opposite-sex pairings, females in same-sex pairs vocalized when closer to a social partner and later in the 30 minutes of social engagement. Remarkably, we found that female mice exhibited no immediate changes in acceleration (movement) to male-emitted vocal signals. Both males and females, in contrast, modulated their behavior following female-emitted vocal signals in a context-dependent manner. Thus, our results suggest female vocal signals function as a means of ultrashort-range communication that shapes mouse social behavior.

## Introduction

Acoustic signaling is a vital means of both intra- and inter-species communication across the animal kingdom, allowing the transfer of information without limitations of light availability or physical proximity between individuals^1,2^. Unlike other communication modalities, acoustic communication is effective over a wide range of distances, and vocalizations are often grouped into two categories: short- and long-range signals^3^. Short-range sounds are emitted by most species that vocalize (e.g., marmosets^4^, rats^5^, and moths^6^), often used for interpersonal communication and to promote social cohesion^7,8^. Long-range signals, while not ubiquitous, are common across the animal kingdom (e.g., wolves^9^, whales^10^, and birds^11^). These signals are generally used to warn others, communicate with distant members, or indicate territoriality^9,12,13^. Research on long-distance calling often focuses on males^14,15^, even though many species show long-range calling from both sexes^16–19^. In some species, females actually emit more long-distance calls than males^20,21^. Female elephants, for instance, are more vocal than males and emit long-range calls to communicate with social partners^22^. These signals are believed to facilitate social recognition over great distances^23^. However, in many animal species the function and range of female-emitted signals is less clear.

In mice, the propagation and behavioral impact of female-emitted signals is less established. Adult mice (*Mus musculus*), while predominately silent in isolation^24^, emit ultrasonic vocalizations, signals spanning 30–110 kHz in frequency^25^, during aggressive and affiliative behaviors^26–30^. Females vocalize during same- and opposite-sex interactions^26,30–34^, while males typically vocalize during opposite-sex interactions^35^. While the exact function of mouse vocalizations has been difficult to elucidate, there are many theories about their role. Male-emitted signals in opposite-sex contexts are believed to help determine potential sexual partners, keep females close, encourage mating, convey social status, and facilitate recognition of other individuals^26,29,36–43^. Female-emitted signals in opposite-sex contexts, in contrast, are believed to signal receptivity^32^. In same-sex interactions, female signals are believed to be more versatile. For example, these signals are proposed as a measure of sociability, social preference, social memory, or social cohesion^27,44,45^. However, despite these proposed functions of mouse vocalizations, the exact role and range of adult ultrasonic vocalizations, specifically female-emitted signals, remains unclear^46^. These uncertainties arise because mice produce no distinct visual cues when vocalizing, making it difficult to determine which mouse emitted individual vocal signals and, importantly, how these signals change the behavior of a social partner. This limits our understanding of mouse vocal communication, because knowing which mouse emits individual signals is critical for determining the biological basis of mouse vocal communication and the role that adult-emitted signals play in shaping social behavior.

Therefore, to overcome this issue, we have implemented an eight-channel microphone array system^47^, allowing us to localize the source of ultrasonic vocal signals. This technology, in conjunction with video tracking software^48^, allows us to track the social and vocal behavior of individual animals over time. Here, we provide a thorough quantitative description of the vocal activity of adult mice and subsequent behavioral responses to vocal emissions, specifically females, during unrestricted dyadic social interactions between a female and either a single male or female social partner. Our results indicate that female mice vocalize later during same-sex social engagements and that mice of both sexes behaviorally respond to female-emitted vocal signals in specific contexts. Strikingly, we revealed that female mice vocalize specifically while in close proximity to other mice, indicating that female-emitted ultrasonic vocal signals may be a means of ultrashort-range communication.

## Results

### Temporal dynamics of vocal expression

**Figure 1:**
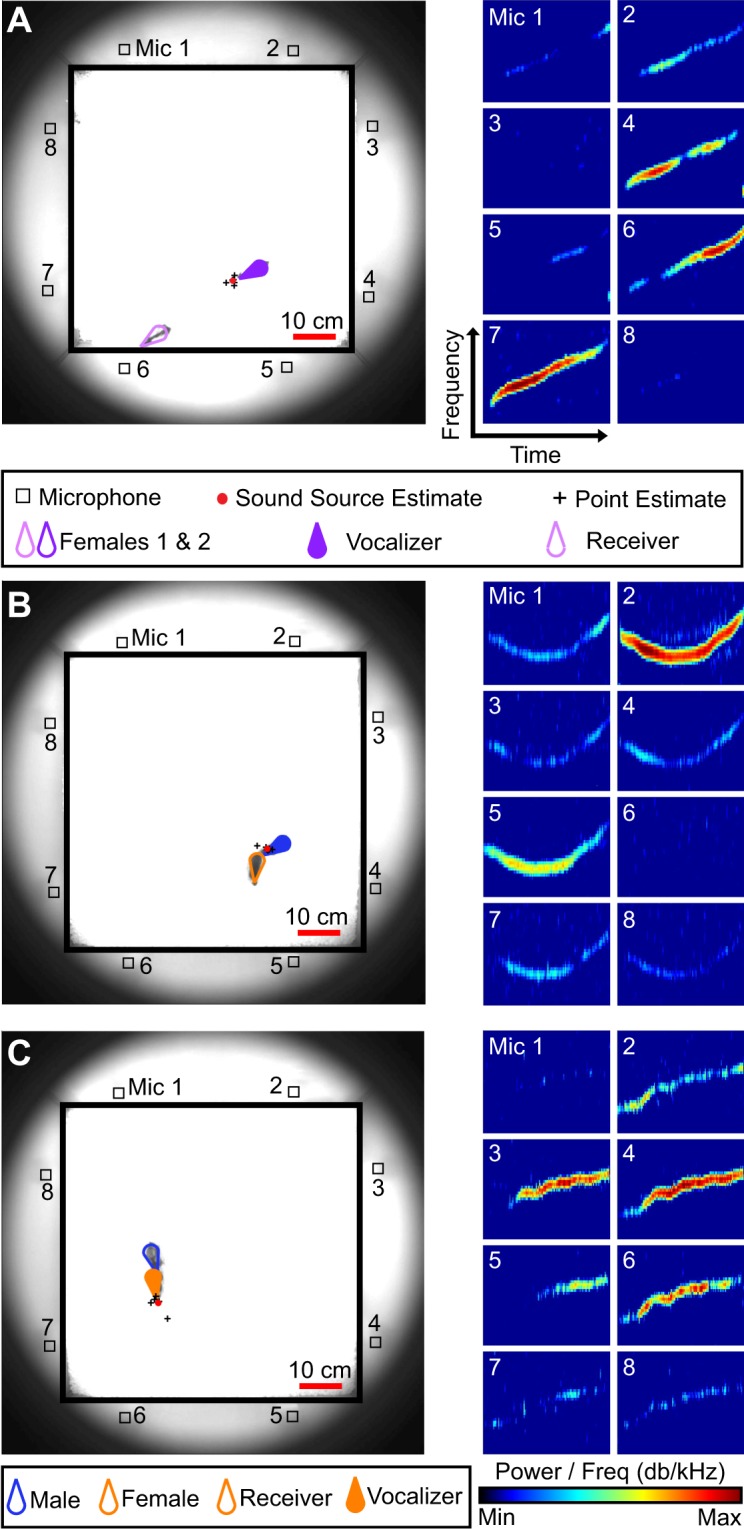
System for localizing sound identifies individual vocalizers during dyadic social interactions. Vocal signals assigned to individual mice in same-sex (**A**) or opposite-sex (**B**,**C**) social contexts. Position of mice (photographs, left) is shown at the time of vocal emission (spectrograms, right).

**Figure 2:**
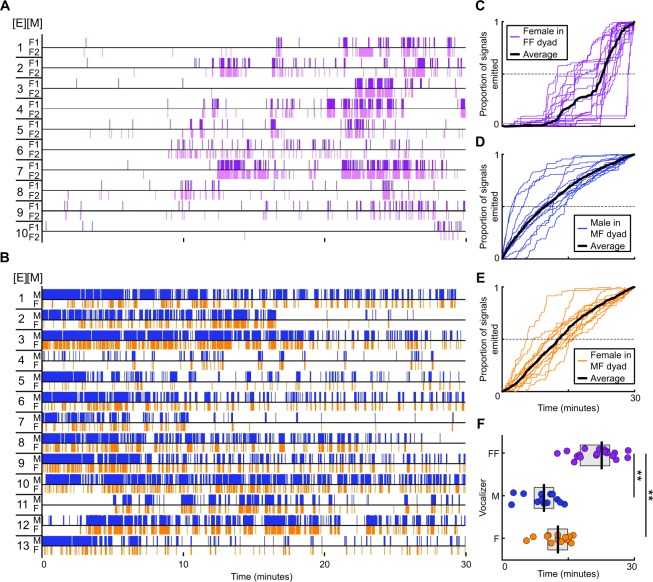
Females vocalize later in same- than opposite-sex contexts. Temporal profile of vocal emission in same-sex (**A**) and opposite-sex (**B**) social contexts. Each vertical line indicates one vocal signal emitted by an individual mouse [M] within an experiment [E]. (**C**–**E**) Cumulative density plots showing the temporal profile of each mouse’s vocal activity. Dashed lines bisect distributions at the time when the mouse had emitted 50% of their total vocal signals. (**F**) Dot plots quantifying temporal differences in vocal emission. Each dot represents the time at which a single mouse had emitted 50% of their total vocal signals; thick vertical lines = median; gray boxes = IQR. **p < 0.01.

A sound source localization system was used to track the vocal behavior of individual mice during unrestrained social interactions (Fig. 1). Two different social contexts, both of which promote vocal emission, were examined. In context 1, a female was paired with another female (same-sex context). Since males rarely vocalize when paired with another male^49,50^, they were not included in the same-sex context. In context 2, a female was paired with a single male (opposite-sex context). We found that all recorded mice vocalized (Fig. 2A,B; range = 10–4,471 signals), but interestingly, the timing of emission differed between social contexts (Fig. 2C–E). Females in same-sex contexts vocalized significantly later than either male or female mice in opposite-sex contexts (Fig. 2F; Kruskal-Wallis, df = 2, chi square = 31.5, p < 10^−5^). Females took 22.5 minutes to emit half of their total signals (IQR = 17.7–24.4 minutes), compared to 9.5 and 12.7 minutes for males and females in an opposite-sex context, respectively (IQR: male = 7.2–11.7 minutes; female = 10.4–14.9 minutes). Together, these results indicate that the temporal dynamics of vocal emission differ across sex, as well as across social condition.

### Social dynamics of vocal expression

**Figure 3:**
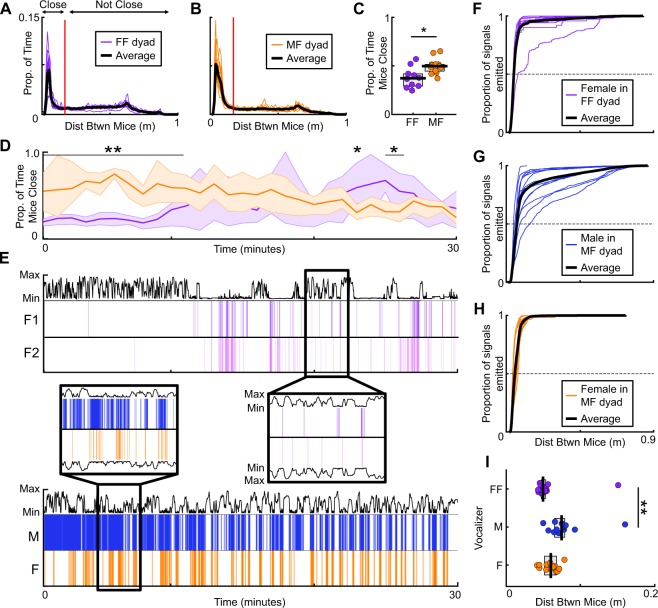
Female mice vocalize in close physical proximity to female conspecifics. Histogram shows the distances between mice during same-sex (**A**) and opposite-sex (**B**) social contexts. (**C**) Proportion of time that each pair of mice spent in close proximity (<20 cm; red line in A and B); dots represent pairs of mice; thick horizontal black line indicates group median; gray box shows IQR. (**D**) Proportion of each minute that pairs of mice spent in close proximity. Line = median; shaded region = IQR. Asterisks indicate significantly different proportions of time in close proximity during each one minute time interval. (**E**) Vocal emission over time during same-sex (purple; top) and opposite-sex (blue/orange; bottom) social contexts. Vocalizer identity labeled to the left (same-sex: F1 = Female 1; F2 = Female 2—arbitrarily numbered; Opposite-sex: M = Male; F = Female). Black trace shows distance between mice over time. Magnified insets (center) show vocal emission and the corresponding distance between mice. Distance traces above and below vocal emission are mirror images. (**F**–**H**) Cumulative density plots of the distance between mice during vocal emission. Dashed lines bisect each distribution at median distance between mice. (**I**) Median distance at which each mouse vocalized; thick vertical lines = median; gray boxes = IQR. **p < 0.01, *p < 0.05.

Mice vocalize primarily while in close physical proximity to conspecifics^51,52^. Thus, the time that animals spend close together may underlie the temporal differences in vocal emission across the two social contexts. We therefore calculated the distance between mice in each frame of video. Mice spent more time in close proximity to each other (within 20 cm) when interacting with an opposite- than same-sex partner (Fig. 3A–C; Mann-Whitney, ranksum = 69, p < 0.01). Specifically, opposite-sex pairs spent significantly more time together than same-sex pairs over the first 10 minutes of social engagement (Fig. 3D; Mann-Whitney, all ranksum >80, all p < 0.05).

When assessing the relationship between vocal emission and the relative distance between mice, we uncovered a striking pattern (Fig. 3E). In same-sex pairs, females vocalized when separated by short distances (Fig. 3F; median = 4.5 cm, IQR = 4.3–4.8 cm). In opposite-sex pairs, however, both males and females vocalized while further away from each other (Fig. 3G,H; males: median = 6.7 cm, IQR = 5.9–7.1 cm; females: median = 5.4 cm; IQR = 4.6–6.1 cm). Males in fact vocalized at distances that were significantly greater than the distances between pairs of vocalizing females (Fig. 3G; Kruskall-Wallis, df = 2, chi square = 18.7, p < 10^−3^, dunn post hoc). This evidence suggests that the ultrasonic vocalizations of female mice represent ultrashort-range communication signals.

### Behavioral responses to vocal signals

Establishing that mouse vocalizations are communicative requires linking vocal emission to changes in behavior. We therefore quantified instantaneous changes in speed (acceleration) in response to vocal emission as a proxy for behavior^32^. Vocal signals used in the analyses had to meet two criteria. First, signals had to be temporally isolated, allowing us to directly quantify the behavioral responses to individual vocal signals. Second, only signals emitted while mice were within 20 cm of a conspecific were included, as these signals are believed to be social signals^34,53^. The speed patterns of the receiving (non-vocalizing) mouse around vocal signals that met these requirements were termed vocal trajectories, with each trajectory spanning 367 ms. To ensure that speed patterns were directly linked to vocal emissions, our control analyses used speed patterns of the same (receiving) mouse over periods of time when no vocal signal was emitted by either mouse. These speed patterns were termed non-vocal trajectories. The first 200 ms of each non-vocal trajectory was speed matched to the first 200 ms of a vocal trajectory, corresponding to the time prior to and including vocal emission. This allowed us to compare post-vocal accelerations to determine whether speed changes of the receiver were specific to vocal emissions or an inherent feature of specific patterns of movement. Lastly, because signal emission may differ based on behavioral context^34,53,54^, all trajectories were separated based on the social context, vocalizer, and speed of the vocalizer relative to the receiver. Together, this analysis allowed us to determine the extent to which mice alter their behavior following vocal emission while controlling for the general patterns of movement prior to vocal emission.

**Figure 4:**
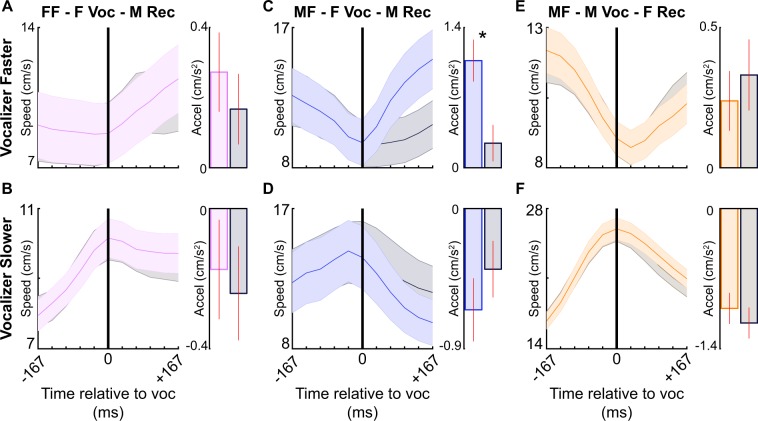
Behavioral responses to temporally isolated vocal signals. Speed of the receiving (non-vocalizing) mouse (indicated with colored lines; vocal trajectory) before and after temporally isolated vocal emissions (black vertical line; time = 0). Gray lines show control speeds without vocal emission (non-vocal trajectory), with speeds before time 0 matched to a vocal trajectory. Shaded area indicates SEM. Bar plots to the right display average acceleration (change in speed) after vocal emission; vertical red line shows SEM. Signals were separated based on the speed of the vocalizer at the time of vocal emission (vocalizer faster than receiver: **A,C,E**; vocalizer slower than receiver: **B**,**D**,**F**). *p < 0.05. FF = same-sex; MF = opposite-sex; F Voc = female vocalizer; M Voc = male vocalizer; F Rec = female receiver; M Rec = male receiver.

We found no difference between vocal and non-vocal trajectories in the female-female condition whether the vocalizer was traveling faster than the receiver (Fig. 4A; Table 1) or slower (Fig. 4B). This indicates that females do not display an immediate behavioral response to the vocal emissions of other females. However, when a female vocalized in the male-female condition while traveling faster than the male, males accelerated significantly more quickly than when no vocal signal was emitted (Fig. 4C). When the female was instead traveling slower than the male, the males’ acceleration was similar regardless of whether the female was silent or vocal (Fig. 4D). Similarly, there were no differences in female acceleration between vocal and non-vocal trajectories in the male-female context regardless of relative speed (Fig. 4E,F). This evidence implies that in specific contexts mouse vocal signals are communicative and change the behavior of the receiving animal.

Given that experiment-wise error rate (i.e., the likelihood of making a type I error or rejecting a true null hypothesis) increases when conducting a series of significance tests^55^, we wanted to control for potentially spurious significance in the analyses. We therefore employed a random sampling procedure. In each context where we found a significant behavioral response to vocal emission (Fig. 4B) we selected a subsample of vocal trajectories that was 25 percent of the total number of examples (n = 31). We then calculated the average difference in acceleration between the subset of vocal and speed-matched non-vocal trajectories (vocal minus non-vocal). This process was repeated 1000 times to generate a distribution of difference values. If there was not a consistent difference between the vocal and non-vocal accelerations, we would expect the center of the distribution to fall near zero. If instead the accelerations of vocal and non-vocal subsamples were dissimilar, we would expect zero to fall outside the distribution. When randomly sampling from opposite-sex contexts in which the vocalizing females were moving faster than the male (Fig. 4B), the males’ acceleration was consistently quicker after females vocalized compared to periods of silence (Supplementary Fig. 1; random sample, p < 0.05). This analysis confirms that mouse vocal signals are communicative and change the behavior of the receiving animal.

**Figure 5:**
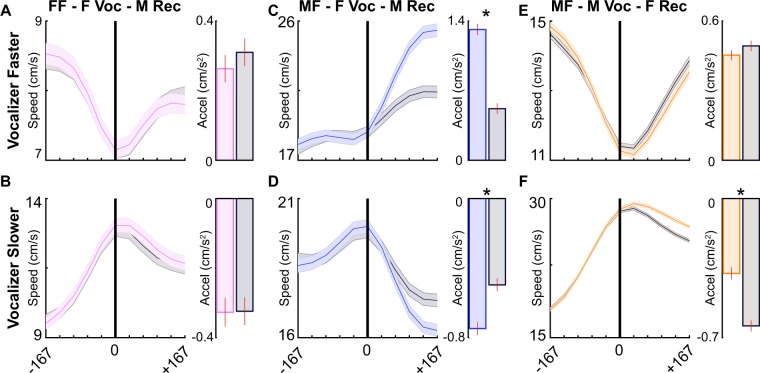
Behavioral responses to all signals emitted by a social partner. Speed of the receiving (non-vocalizing) mouse (represented with colored lines; vocal trajectory) before and after every vocal signal emitted by a social partner (black vertical line; time = 0). Gray lines show control speeds without vocal emission (non-vocal trajectory), with speeds before time 0 matched to a vocal trajectory. The shaded area indicates SEM. Bar plots to the right display average acceleration (change in speed) after vocal emission; vertical red line shows SEM. Signals were partitioned based on the speed of the vocalizer at the time of vocal emission (vocalizer faster than receiver: **A,C,E**; vocalizer slower than receiver: **B,D,F**). *p < 0.05. FF = same-sex; MF = opposite-sex; F Voc = female vocalizer; M Voc = male vocalizer; F Rec = female receiver; M Rec = male receiver.

Temporally isolated signals are beneficial for directly quantifying behavioral responses to vocal emission; however, mice often emit multiple vocal signals in quick succession^25^. Therefore, temporally isolated signals may represent a specific subset of signals that are atypical of mouse vocal emission. To control for this possibility, we replicated the previous analysis, but included signals emitted in close temporal succession (Fig. 5; Table 2). We again found that females are unresponsive to female-emitted signals (Fig. 5A,B). In the opposite-sex condition, however, males consistently altered their behavior in response to a female-emitted signal. When females were traveling faster than the males, males accelerated more quickly in response to a vocal than silent female (Fig. 5C). When females were traveling slower than the males, males decelerated more rapidly in response to a vocal than silent female (Fig. 5D). When the male vocalized, females responded in a context-dependent manner (Fig. 5E,F). Females were unresponsive to male-emitted vocal signals when the male was traveling faster than the female (Fig. 5E). However, when males were traveling slower than the females, the females decelerated less quickly in response to vocal than silent males (Fig. 5F). Together, our results indicate that mice directly respond to innate vocal signal emission in a context-specific manner.

When assessing the behavioral response to vocal emission, we found a single context in which mice respond to temporally isolated signals and multiple contexts in which mice respond to non-isolated signals. Because the likelihood of making a type I error also increases with larger sample sizes^56,57^, we wanted to control for potentially spurious significance in the analyses using all vocal signals. Here, we conducted another random sampling procedure in which a subsample of vocal trajectories was selected. The number of subsamples selected was size-matched to the number of temporally isolated signals emitted in the same context (Fig. 5C,D,F). When randomly sampling from opposite-sex contexts in which the vocalizing females were moving faster than the male (Fig. 5C), the males’ acceleration was consistently quicker after females vocalized compared to periods of silence (Supplementary Fig. 2A; n = 48 random sample, p < 0.05). Interestingly, this was the only context in which we found a significant behavioral response to temporally isolated signals. When randomly sampling from opposite-sex contexts in which vocalizing females were moving slower than the male (Fig. 5D) or in which vocalizing males were moving slower than the female (Fig. 5F), there were no significant differences (Supplementary Fig. 2B,C; n = 87, 290, respectively; random sample, all p values > 0.05). These results indicate that female vocalizations are associated with robust changes in male behavior in specific behavioral contexts.

### Behavioral responses to vocal signals change over time

**Figure 6:**
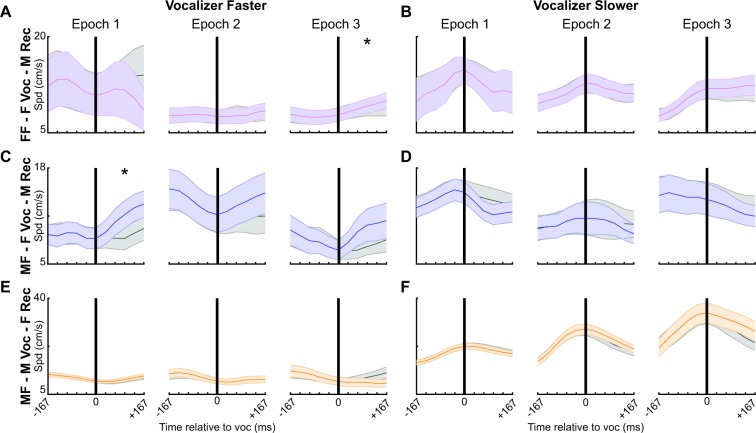
Behavioral responses to vocal emission change over time. Speed of the receiving mouse (shown with colored lines; vocal trajectory) before and after temporally isolated vocal emissions (black vertical line; time = 0). Gray lines denote control speeds without vocal emission (non-vocal trajectory), with speeds prior to time 0 matched to a vocal trajectory. The shaded area indicates SEM. Recordings were segmented into three bins: the first (Epoch 1), second (Epoch 2), or final ten minutes (Epoch 3). Signals were separated based on the speed of the vocalizer at the time of vocal emission (vocalizer faster than receiver: **A,C,E**; vocalizer slower than receiver: **B,D,F**). Acceleration (change in speed) after time 0 was compared between the vocal and non-vocal trajectories. *p < 0.05. FF = same sex; MF = opposite sex; F Voc = female vocalizer; M Voc = male vocalizer.

Because mice alter their responses to vocalizations broadcast from a speaker over time^39^, we thought that a similar phenomenon might occur during natural behavior. To quantify this, we separated recordings into ten-minute periods, producing three unique epochs that were each independently analyzed. For each ten-minute period, all speed trajectories were again separated by social context, vocalizer, and speed of the vocalizer (Fig. 6; Table 3). Since the movement patterns of mice change over time^58^, all speed-matched non-vocal trajectories were selected from the same time bin. We found that in same-sex contexts females did not respond to vocal emissions within the first 20 minutes of social engagement, regardless of which female was traveling faster (Fig. 6A,B). However, when the vocalizer was traveling faster in the final ten minutes, the receiving female accelerated more rapidly following vocal emissions than in periods of silence (Fig. 6A). However, following a random sampling procedure (Supplementary Fig. 3A; n = 72), we found no significant differences (p > 0.05), challenging the robustness of the finding. In the final ten minutes when the vocalizing female was traveling slower (Fig. 6B), the speed of the receiving female was unaffected by vocal emission.

**Table 1:** Acceleration comparisons between vocal and non-vocal trajectories for temporally isolated signals in each behavioral context.

	Relative Speed of Vocalizer	Vocal Trajectory		Non-Vocal Trajectory		t	p
		Mean Accel	Std Accel	Mean Accel	Std Accel		
FF - F Voc - F Rec	Faster	0.27	1.34	0.17	1.19	0.71	0.48
	Slower	−0.15	1.38	−0.21	1.30	0.37	0.71
MF - F Voc - M Rec	Faster	1.07	1.45	0.25	1.26	2.89	0.01*
	Slower	−0.65	1.89	−0.39	1.70	0.90	0.37
MF - M Voc - F Rec	Faster	0.21	1.75	0.30	2.08	0.55	0.59
	Slower	−1.00	2.63	−1.14	2.63	0.67	0.50

Units = cm/s^2^, *significant differences.

**Table 2:** Acceleration comparisons between vocal and non-vocal trajectories for all signals in each behavioral context.

	Relative Speed of Vocalizer	Vocal Trajectory		Non-Vocal Trajectory		t	p
		Mean Accel	Std Accel	Mean Accel	Std Accel		
FF - F Voc - F Rec	Faster	0.16	1.24	0.19	1.32	−0.85	0.39
	Slower	−0.25	1.57	−0.24	1.50	−0.05	0.96
MF - F Voc - M Rec	Faster	1.31	2.48	0.52	2.36	2.07	0.04*
	Slower	−0.75	2.30	−0.50	2.23	2.43	0.02*
MF - M Voc - F Rec	Faster	0.45	2.64	0.49	2.69	−1.33	0.18
	Slower	−0.38	3.20	−0.64	2.93	6.60	<0.01*

Units = cm/s^2^, * = significant differences.

**Table 3:** Acceleration comparisons between vocal and non-vocal trajectories in each behavioral context over time.

	Relative Speed of Vocalizer	Bin	Vocal Trajectory		Non-Vocal Trajectory		t	p
			Mean Accel	Std Accel	Mean Accel	Std Accel		
FF - F Voc - F Rec	Faster	1	−0.46	1.07	0.59	0.34	−2.47	0.07
		2	0.17	0.19	0.09	0.27	0.48	0.66
		3	0.44	0.05	0.18	0.20	3.05	0.04*
	Slower	1	−0.71	0.68	−0.57	0.45	−0.78	0.48
		2	−0.33	0.27	−0.28	0.30	−0.43	0.69
		3	0.10	0.11	−0.09	0.17	5.70	0.00*
MF - F Voc - M Rec	Faster	1	1.28	0.36	1.10	1.02	2.72	0.02*
		2	0.81	0.00	1.63	1.64	1.93	0.07
		3	1.11	0.38	1.64	1.05	0.24	0.81
	Slower	1	−0.73	0.52	1.59	1.35	−0.55	0.59
		2	−0.58	0.26	2.31	1.87	−0.72	0.48
		3	−0.62	0.37	1.85	1.88	0.18	0.85
MF - M Voc - F Rec	Faster	1	0.38	0.29	1.76	1.84	0.47	0.64
		2	0.14	0.07	1.83	1.67	0.29	0.77
		3	−0.18	0.65	1.51	3.07	−1.85	0.07
	Slower	1	−0.55	0.53	1.72	2.09	−0.11	0.91
		2	−1.50	1.62	2.96	2.96	0.46	0.64
		3	−1.38	2.19	3.98	2.97	1.16	0.26

Units = cm/s^2^, *significant differences.

We also quantified responses to vocal emission over time in opposite-sex social contexts. Males responded to female-emitted vocal signals in a single context: during the first 10 minutes while the female was traveling more rapidly than the male (Fig. 6C; Supplementary Fig. 3B; n = 17). Males were unresponsive to a female-emitted signal while the female was traveling slower than the male (Fig. 6D). Female mice were unresponsive to male-emitted vocal signals, regardless of context (Fig. 6E,F). In sum, these results indicate that female vocal emission alters the behavior of male mice during the initial stages of a social experience, but surprisingly, female mice do not instantaneously change their acceleration in response to male vocal signals in any context.

## Discussion

Implementing a sound-source localization system provided unprecedented access into the vocal behavior of individual adult mice during social interactions. We found that laboratory-bred female mice vocalize during dyadic interactions regardless of social context, corroborating previous findings^31,32,44,59^. Interestingly, we uncovered two novel features of female vocal emission. First, females vocalize and spend more time with a social partner later in same- than opposite-sex pairings. Second, females almost exclusively vocalize in close proximity to other mice, which contrasts starkly with the broad range of social distances at which males produce vocal signals. These findings suggest that, while ultrasonic vocal signals are generally used during close-range communication, laboratory-bred female mice specifically use vocal emission as a means of ultrashort-range communication. If female vocalizations are a mechanism of ultrashort-range signaling, this may explain the longer latency to vocalize in same-sex settings, as pairs of females spend less time in close proximity at the beginning of a recording than mixed-sex pairs. The most compelling finding, however, is that mice behaviorally respond to the vocal emissions of other mice. Specifically, when a female mouse vocalizes while traveling faster than a male partner, the male accelerates. In fact, the responses depend on the timing of vocal emission, with males only reacting in the first 10 minutes of a recording. Therefore, our results indicate that female-emitted vocal signals change the behavior of social partners. Moreover, male and female mice emit ultrasonic vocal signals to communicate over differing distances, with females using the signals as a means of ultrashort-range communication.

Ultrasonic signals propagate inefficiently over long distances, as they are quickly attenuated by the environment and easily impeded by small objects^60^. To overcome the challenges of propagation, some features of male vocal signals may facilitate communication over greater distances. Heckman *et al*.^31^, for instance, found that male-emitted signals in opposite-sex contexts have significantly lower mean frequencies, which may allow the signals to propagate through the environment more efficiently than female-emitted signals. However, other studies in both laboratory-bred^34^ and wild-bred mice^46,61^ found no differences between the low frequencies of male and female signals, indicating that further research is necessary to assess the spectrotemporal properties of mouse ultrasonic vocal signals and how these signals differ across both sex and social contexts.

Why male and female mice communicate over differing distances is an open question with several potential explanations. The reliance of mice on ultrasonic vocalizations may be a means to avoid eavesdropping by predators^60^. Thus, the reliance of female mice on shorter-range signals may be further protection against eavesdropping. While this is an interesting possibility, we believe alternative explanations are more likely for multiple reasons. First, in a wild-bred mouse population, which we posit would be more attuned to predation, female mice vocalized at greater distances from social conspecifics^44^. Second, many natural predators of the mouse can detect ultrasonic frequencies. Cats and dogs, for instance, perceive sounds up to 85 and 47 kHz^62,63^. An intriguing alternative explanation is that this ultrashort-range emission observed in females is the standard means of communication for mice, and males instead alter their signals to enhance the range of acoustic propagation. Male signals across the animal kingdom are often used as broadcast or advertisement calls, indicating territoriality or fitness^64–67^. Male flies, for instance, will alter the intensity of their courtship song to broadcast to every visible female^68^. Thus, it may be evolutionarily advantageous for male mice to alter their vocal emissions to propagate greater distances, as this adaptation may enhance the likelihood to sire offspring.

Our finding that females vocalize exclusively when in close physical proximity may indicate that females rely upon other social cues to elicit vocal signals. For example, specific tactile or urinary pheromonal cues, utilized in short range signaling, may trigger female vocal production. In support, different populations of neurons are activated in the mouse accessory olfactory systems of male and female mice upon pheromonal stimulation^69^. Furthermore, experience dependent plasticity regulates the differential expression of pheromone-sensing neurons in males and females^70^, and female mice deficient for Trpc2 receptors, which are used to detect pheromones, display vocal patterns characteristic of males^71^. Interestingly, however, urine alone is not sufficient to elicit vocalizations from female mice^72^, whereas male mice reliably vocalize in response to female scent cues^28,73^. An intriguing explanation for this difference is that females, unlike males, may require multiple different sensory modalities to elicit vocal activity (see^44^). Female vocal emission would not be the only mouse behavior relying upon multiple modalities, as other behaviors have been shown to require multiple sensory cues (e.g., pup-directed aggression from adult males^74^ and pup-retrieval by mothers^75,76^). Clearly, future studies will be necessary to directly determine the neural and social mechanisms gating female vocal production.

Contrary to previous work^24^, we found that females vocalize later in same- than opposite-sex contexts. This discrepancy can potentially be attributed to two critical points. First, we found that females vocalize almost exclusively while near another female. Second, the area of our recording arena is ~5806 cm^2^ (76 × 76 cm). Given the large area, which allowed mice to move freely without encountering a social partner, females could initially spend more time further apart, thus limiting their propensity to vocalize. Interestingly, every female vocalized in our recordings. This too contrasts with previous work, which showed that females vocalized in only 66–81% of same-sex interactions lasting less than 5 minutes^24,33^. Our longer-duration recordings may have provided more opportunity for female mice to directly interact with a social partner. Consequently, we revealed that female mice produce ultrasonic signals capable of affecting the dynamics of social interactions.

Communication shapes social dynamics across the animal kingdom, allowing individuals to warn groups about predators^77^, display reproductive fitness^78^, or encourage affiliative behaviors^79^. In mice, male ultrasonic vocalizations are thought to encourage female approach^39,80^ or signal social status^49,81^. Female ultrasonic vocalizations emitted during same-sex interactions are believed to have many functions, including playing a role in dominance hierarchy formation^33^, functioning as territorial calls^46^, facilitating cooperative behaviors^59^, indexing sociability^27^ or familiarity^27,44,82^, and denoting motivational state^45^. Emerging evidence also indicates that females vocalize in mixed-sex contexts^31,32,34^. However, the inability to determine which mouse in a group emits individual vocalizations has impeded progress towards determining the function of female vocal emissions across social contexts. Our results provide direct evidence that female-emitted signals are sufficient to alter the behavior of a social partner, thus playing an undeniable role in regulating complex social interactions.

Vocal signals are context dependent, even within a particular social situation. Mice of both sexes alter the acoustic features of vocal signals across behavioral^34^ and social contexts^53,54^. The meaning of specific vocal alterations, however, remains unknown. Some animals (e.g., lemurs^83^ and bats^84^), emit distinct types of vocalizations with specific meanings. While the meaning of specific types of mouse vocalizations is unclear, females may emit specific types of signals during distinct types of social encounters. This could explain why both males and females modulate their activity in response to female-emitted vocal signals, but only in discrete behavioral contexts. Alternatively, the response of mice to vocal signals may depend upon behavioral or motivational state, as responses to female vocalizations were context-specific (e.g., when the vocalizing female was traveling faster). Disentangling the interplay between vocal repertoire and behavioral state will be essential to fully understanding the function of mouse vocal communication.

## Materials and Methods

### Subjects

Mice (n = 10 male; n = 20 female; aged 9–22 weeks) of a B6.CAST-Cdh23Ahl +/Kjn background were raised in the Life Science Research Facility at the University of Delaware. At three weeks of age, mice were group-housed with same sex littermates in cages containing 4 or fewer animals. All cages contained ALPHA-dri bedding, environmental enrichment, and animals were allowed *ad lib* access to food and water. Mice were implanted with a light-activated microtransponder for identification. At least two weeks before experiments began, mice were isolate housed, as group housing has been shown to alter social behavior^85–87^. The colony room was kept on a 12/12 dark/light cycle (lights on at 9 pm). All experiments were conducted during the dark phase. Experiments were conducted in accordance with the Guide for the Care and Use of Laboratory Animals of the National Institutes of Health and approved by the University of Delaware Animal Care and Use Committee (protocol number: 1275-2017-0).

### Experimental Setup

Mice were recorded in either an opposite-sex (one male and one female; n = 13; see^34^), or same-sex (two females; n = 10) social context. At least two days before experiments, mice were marked with blonde hair dye^48^. In the opposite-sex condition, males received a five-dot pattern and females were unpainted. For same-sex pairs, all females were painted to avoid differential experiences. Patterns included five dots, two vertical lines, two horizontal lines, or a slash. If the dye faded, mice were repainted with the same pattern.

Mice received unrestrained exposure to an adult animal of the opposite sex one day after dying their hair^34^, as previous opposite-sex experience enhances vocal activity^72,88^. Exposure sessions were terminated by an experienced observer prior to successful copulation or after 10 minutes. Stimulus mice were never used as subjects.

Prior to a potential experiment, vaginal lavage was used to determine the female’s reproductive state^34^. Cells were placed on a slide, stained with crystal violet, and imaged. If the majority of cells were cornified epithelial cells without a nucleus^89^, females were considered to be in estrus and recorded. For same-sex recordings, both females were required to be in estrus.

In each recording, two mice interacted for 30 minutes. Females and males were used no more than four and two times, respectively. To control for previous social experience, mice were recorded only once with the same social partner. Females experiencing successful copulation (male falling over after an extended period of mounting, n = 2) were removed from subsequent recordings.

Video and audio data were concurrently recorded in an anechoic chamber. Audio data was sampled by an 8-channel microphone array (microphones from Avisoft-Bioacoustics; Glienicke, Germany; CM16/CMPA40-5V) at 250,000 Hz (National Instruments; Austin, TX; PXIe-1073, PXIe-6356, BNC-2110) and low-pass filtered at 200 kHz (Krohn-Hite, Brockton, MA; Model 3384). Video data was recorded by a single camera (FLIR; Richmond, BC; GS3-U3-41C6M-C) above the arena using BIAS software (https://bitbucket.org/iorodeo/bias/downloads/). A counter pulse triggered the camera to sample at 30 Hz and facilitated alignment of the audio and video recordings. Recording devices were controlled with custom-written Matlab software.

Recordings were conducted in a large arena (~5806 cm^2^; 76 × 76 cm) as described previously^34^. Briefly, the floor of the arena was covered in a layer of ALPHA-dri bedding. Before each recording, two 15 second tests were conducted. First, a string of LEDs surrounding each of the eight microphones was used to determine microphone positions. Next, camera focus was confirmed after overhead infrared lights were turned on. The infrared lights remained on for the duration of a dyadic recording (30 minutes). Finally, two ten-minute recordings, each containing a single mouse from the dyadic recording, were conducted.

### Data processing

Audio and video data were processed with custom-written software (Matlab) on the University of Delaware’s high-performance computer cluster.

### Tracking

Mouse position across recordings was automatically tracked with the Motr program^48^ (http://motr.janelia.org). Tracking was confirmed by manual inspection.

### Audio segmentation

Vocal signals were extracted using a multi-taper spectral analysis (Ax^54^). Data from each microphone was bandpass filtered (30–110 kHz) and Fourier transformed with multiple discrete prolate spheroidal sequences used as windowing functions (K = 5; NW = 3). An F-test was used to determine if individual time-frequency points exceeded background noise (p < 0.05). This was repeated for multiple segment lengths (NFFTs = 64, 128, 256) on each microphone. A single spectrogram was generated by combining all eight audio channels and convolving with a square box to fill in small gaps, and signals were extracted from this average audio trace. The convolving window was 11 by 15 pixels in frequency and time, respectively. Continuous regions containing 1500 or more pixels were considered individual vocal signals.

Because signals discontinuous in frequency or time could consist of unique signals emitted by different mice, any discontinuity marked the delineation between separate signals^34^. The only exceptions were harmonic signals, and only the fundamental frequencies were analyzed. Ax determined the start and stop times and calculated a frequency contour (a series of points in frequency and time) for each signal. Extracted vocal signals were plotted for visual inspection.

### Sound source localization

A sound source localization system was used to assign vocal signals to individual mice^47^. For each signal, the system computed eight individual estimates of where the sound originated. For each estimate, the data from a single microphone was omitted and the estimate was computed from the remaining seven microphones. Each microphone was omitted once for each extracted signal. The x- and y-positions of the eight estimates were then averaged to estimate where the sound originated (sound source estimate). The eight point estimates and the sound source estimate were used to generate a probability density across the cage, indicating the likelihood that the signal came from every possible position in the cage. Each mouse was assigned the probability density value (D) corresponding to the position of their nose (determined from the tracking output of Motr). The two density values were used to compute the probability that the signal was emitted by either animal (Mouse Probability Index: MPI).

$$MP{I}_{n}=\frac{{D}_{n}}{{\sum }_{i=1}^{M}{D}_{i}}$$

where n = mouse number (1 or 2) and M = the total number of mice (2). The MPI value for a single mouse needed to reach 0.95, indicating a 95% likelihood that the signal originated from that mouse.

### Determining speed/acceleration

To exclude non-social signals, signals were only included in analyses when mice were within 20 cm of each other^34^. For each vocal emission, we quantified the speed of the non-vocalizing mouse (receiver) in the five frames before and after signal emission, plus the frame of signal emission (11 total frames, or 367 ms). These periods were defined as a vocal trajectory. Speed patterns of the receiving mouse over periods of time (376 ms) without vocal emissions were classified as non-vocal trajectories. Once all non-vocal trajectories were identified, we found the differences in speed across the 1^st^ 200 ms of a non-vocal and vocal trajectory, corresponding to the time preceding and including the vocal emission. The speed differences were summated to generate the absolute speed difference between the vocal and non-vocal trajectories. Each vocal trajectory was compared to every non-vocal trajectory. The non-vocal trajectory producing the smallest absolute speed difference (i.e., the non-vocal trajectory whose speed profile most closely matched that vocal trajectory) was used as our speed-matched control. Non-vocal trajectories were used only once to prevent oversampling individual trajectories.

To quantify behavior following a vocal emission, we calculated instantaneous acceleration in the 167 ms (or five frames of video) following vocal emission. Changes in speed (acceleration) between each pair of consecutive frames was calculated and these values were averaged to get a single numerical representation of instantaneous acceleration. This was done for each vocal trajectory and each speed-matched non-vocal trajectory. Then, to determine whether acceleration differed as a function of vocal emission, we compared the distribution of instantaneous acceleration values for the vocal trajectories to the distribution for the non-vocal trajectories using a paired t-test. Moreover, behavioral responses to vocal and non-vocal trajectories were separated based on vocalizer (female in same-sex context; male or female in opposite-sex context) and behavior (whether the vocalizer was traveling faster or slower than the receiver). In an effort to ensure that individual trajectories consisted of periods of time containing only one vocal signal, thus allowing us to accurately dissociate pre- and post-vocal activity, the analyses shown in Fig. 4 excluded all signals within 367 ms of another signal. For the analyses shown in Fig. 5, we did not exclude trajectories including multiple vocal signals.

### Random sampling procedure – controlling for sample size

Because large sample sizes are more likely to detect significance even when differences between groups are small^56,57^, we needed to account for sample size in the analyses displayed in Fig. 5, where we used all signals. Therefore, we randomly selected a subsample of vocal (and corresponding non-vocal) trajectories that was size-matched to the number of vocal signals from the temporally isolated analysis in the same condition (Fig. 4). For each individual trajectory, we calculated the average acceleration following the time of vocal emission, as outlined previously. We then found the average acceleration difference between the vocal and non-vocal trajectories (vocal minus non-vocal). This was repeated 1000 times to generate a distribution of acceleration differences. If behavior did not differ as a function of vocal emission, we would not expect that acceleration between the vocal and non-vocal subsamples differ, providing a distribution falling around zero. If instead behavior did differ as a function of vocal emission, we would expect that the distribution would fall to one side of zero, indicating that after the time of vocal emission, the acceleration of mice that had just received a vocal signal was either consistently greater than or less than would be expected if no vocal signal had been emitted.

### Temporal speed differences in specific epochs

To determine how behavior changed over time, we quantified the response to vocal signals after separating the recording into three unique 10-minute epochs. To control for potential behavioral differences across the recording, speed-matched controls were selected from the same 10-minute epoch. All signals used in this analysis were temporally isolated signals.

### Statistics

All statistical tests (alpha set at 0.05) were implemented in Matlab. Nonparametric tests were conducted when sample sizes were below 15 using a Mann-Whitney or Kruskal-Wallis with a Dunn’s post-hoc, and medians and interquartile ranges were used to report central tendency and variability, respectively. Since parametric tests are robust to larger sample sizes^55^, we used parametric tests when samples sizes exceed 15. When statistically analyzing behavioral responses, we used a paired t-test, with means and standard errors representing central tendency and variability, as comparisons were orthogonal (vocal and non-vocal trajectories) and paired (speed matched). In an effort to ensure that individual t-tests were not significant due to chance, we employed a random sampling procedure. For each test, we randomly selected 25% of the vocal (and corresponding non-vocal trajectories) and conducted the analyses as outlined in the Random Sampling Procedure section.

## Supplementary information

Supplementary Information.
