# Supplementary material for: Ultrashort-range, high-frequency communication by female mice shapes social interactions
Source: Sci Rep. 2020 Feb 14;10:2637. doi: 10.1038/s41598-020-59418-0 (PMC7021676; doi:10.1038/s41598-020-59418-0)
Supplement: Supplementary file 1 — Supplementary Information. [file 41598_2020_59418_MOESM1_ESM.pdf]

Supplementary Information

Title:

Ultrashort-range, high-frequency communication by female mice shapes social interactions.

Authors:

Warren, M.R.[1], Clein, R.S.[1], Spurrier, M.S.[1], Roth, E.D.[1], and Neunuebel, J.P.[1 *]

Affiliations:

[1] Dept. of Psychological and Brain Sciences, University of Delaware, Newark, Delaware, USA

***** Correspondence and requests for materials should be addressed to **jneun@udel.edu**.

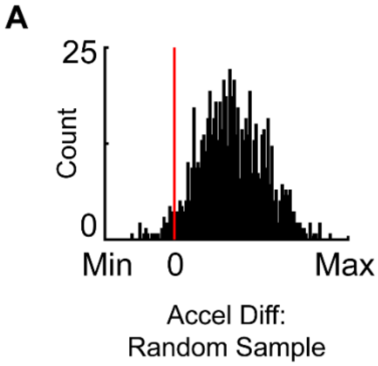

Supplementary Figure 1. Controlling for significance of behavioral response to temporally isolated signals. Corresponding to the data in **Figure 4B**; Bootstrap resampling revealed robust changes in a male's acceleration when the female vocalizer was moving faster. A thousand unique subsamples were generated, with the number of data points per sample equaling 25 percent of the total examples. The acceleration difference between vocal and non-vocal trajectories (vocal minus non-vocal) was calculated for each subsample (black bars show distribution), and the overall distribution of differences was compared to zero (vertical line). Gray line = $p>0.05$; red line = $p<0.05$.

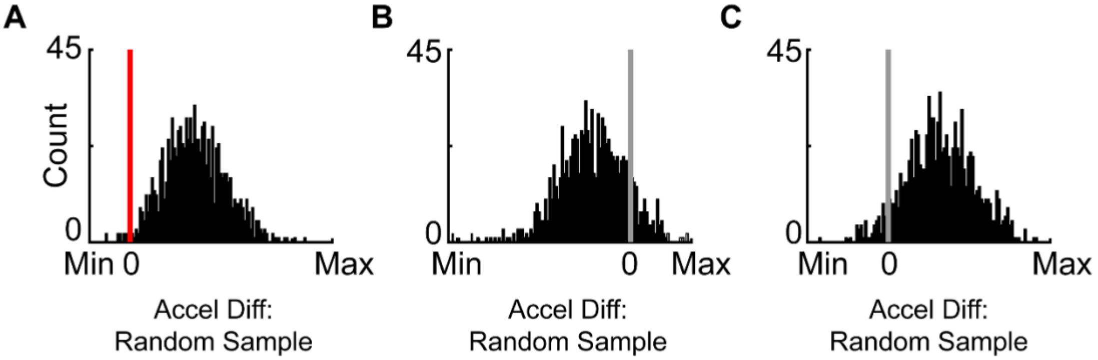

Supplementary Figure 2. Controlling for significance of behavioral response to all vocal signals. A-C, Bootstrap resampling revealed that the significant changes in a receiver's acceleration when the vocalizer was moving faster (**Figure 5C**) persisted regardless of the sample size. In contrast, the significant changes in a receiver's acceleration when the vocalizer was moving slower (**Figure 5D,F**) disappeared when accounting for sample size. A thousand unique subsamples were generated, with the number of data points per sample matching the number of temporally-isolated signals emitted in the same condition. The acceleration difference between vocal and non-vocal trajectories (vocal minus non-vocal) was calculated for each subsample (black bars show distribution), and the overall distribution of differences was compared to zero (vertical line). Gray line = $p>0.05$; red line = $p<0.05$. FF = same sex; MF = opposite sex; F Voc = female vocalizer, M Voc = male vocalizer; F Rec = female receiver, M Rec = male receiver.

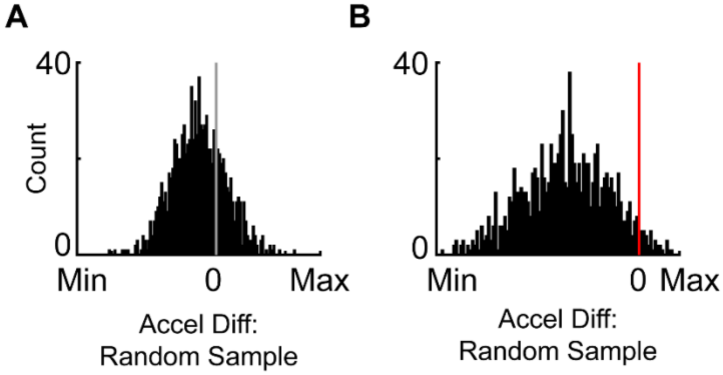

Supplementary Figure 3. Controlling for significance of behavioral response over time. Corresponding to the data in **Figure 6A,C**; Bootstrap resampling revealed no significant changes in a female's acceleration during the last ten minutes (epoch 3) after her female social partner vocalized while traveling faster. However, males showed a robust behavioral response to female-emitted signals during the first ten minutes (epoch 1) while the female traveled faster. A thousand unique subsamples were generated, with the number of data points per sample equaling 25 percent of the total examples. The acceleration difference between vocal and non-vocal trajectories (vocal minus non-vocal) was calculated for each subsample (black bars show distribution), and the overall distribution of differences was compared to zero (vertical line). Gray line = $p>0.05$; red line = $p<0.05$.
